# Nitrate as an alternative electron acceptor destabilizes the mineral associated organic carbon in moisturized deep soil depths

**DOI:** 10.3389/fmicb.2023.1120466

**Published:** 2023-02-08

**Authors:** Wei Song, Chunsheng Hu, Yu Luo, Tim J. Clough, Nicole Wrage-Mönnig, Tida Ge, Jiafa Luo, Shungui Zhou, Shuping Qin

**Affiliations:** ^1^Fujian Provincial Key Laboratory of Soil Environmental Health and Regulation, College of Resources and Environment, Fujian Agriculture and Forestry University, Fuzhou, Fujian, China; ^2^Hebei Provincial Key Laboratory of Soil Ecology, Center for Agricultural Resources Research, Institute of Genetic and Developmental Biology, Chinese Academy of Sciences, Shijiazhuang, Hebei, China; ^3^Zhejiang Provincial Key Laboratory of Agricultural Resources and Environment, Institute of Soil and Water Resources and Environmental Science, Zhejiang University, Hangzhou, China; ^4^Faculty of Agriculture and Life Sciences, Lincoln University, Lincoln, New Zealand; ^5^Faculty of Agricultural and Environmental Sciences, Grassland and Fodder Sciences, University of Rostock, Rostock, Germany; ^6^State Key Laboratory for Managing Biotic and Chemical Threats to the Quality and Safety of Agro-Products, Key Laboratory of Biotechnology in Plant Protection of Ministry of Agriculture and Zhejiang Province, Institute of Plant Virology, Ningbo University, Ningbo, China; ^7^AgResearch Ltd., Hamilton, New Zealand

**Keywords:** nitrate leaching, global warming, greenhouse gas emission, MAOC, deep soil

## Abstract

Numerous studies have investigated the effects of nitrogen (N) addition on soil organic carbon (SOC) decomposition. However, most studies have focused on the shallow top soils <0.2 m (surface soil), with a few studies also examining the deeper soil depths of 0.5–1.0 m (subsoil). Studies investigating the effects of N addition on SOC decomposition in soil >1.0 m deep (deep soil) are rare. Here, we investigated the effects and the underlying mechanisms of nitrate addition on SOC stability in soil depths deeper than 1.0 m. The results showed that nitrate addition promoted deep soil respiration if the stoichiometric mole ratio of nitrate to O_2_ exceeded the threshold of 6:1, at which nitrate can be used as an alternative acceptor to O_2_ for microbial respiration. In addition, the mole ratio of the produced CO_2_ to N_2_O was 2.57:1, which is close to the theoretical ratio of 2:1 expected when nitrate is used as an electron acceptor for microbial respiration. These results demonstrated that nitrate, as an alternative acceptor to O_2_, promoted microbial carbon decomposition in deep soil. Furthermore, our results showed that nitrate addition increased the abundance of SOC decomposers and the expressions of their functional genes, and concurrently decreased MAOC, and the ratio of MAOC/SOC decreased from 20% before incubation to 4% at the end of incubation. Thus, nitrate can destabilize the MAOC in deep soils by stimulating microbial utilization of MAOC. Our results imply a new mechanism on how above-ground anthropogenic N inputs affect MAOC stability in deep soil. Mitigation of nitrate leaching is expected to benefit the conservation of MAOC in deep soil depths.

## Graphical Abstract

Mechanisms of nitrate on deep soil MAOC. 
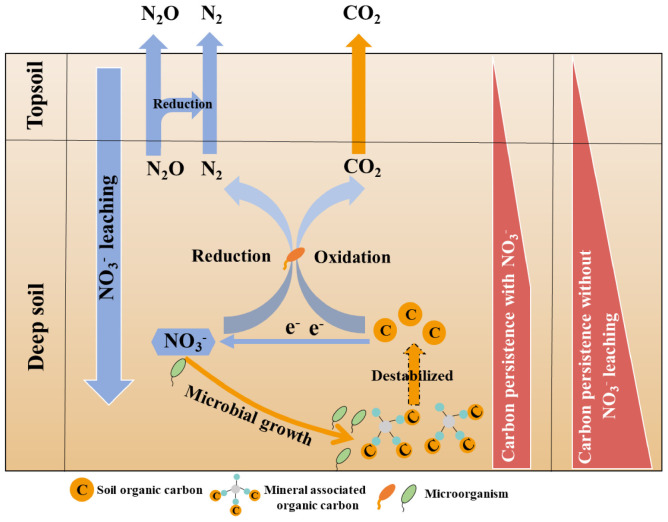

## 1. Introduction

Globally, the stock of soil organic carbon (SOC) is estimated to be as high as 2300 Pg in the 3 m depth, which is about 3-fold the size of the atmospheric carbon dioxide (CO_2_) pool (770 Pg) (Lal, 2004). The annual CO_2_ emissions due to soil respiration are reported to range from 60 to 100 Pg C yr^–1^, which is an order of magnitude greater than current fossil fuel CO_2_ emissions (Bond-Lamberty and Thomson, 2010; Oertel et al., 2016; Xu and Shang, 2016) and account for 5∼25% of total annual CO_2_ emissions globally (Raich and Potter, 1995; Wang et al., 2018). CO_2_ is the dominant greenhouse gas and the atmospheric concentration of CO_2_ has increased from 277 μl l^–1^ in 1750 to 411 μl l^–1^ in 2019 (Friedlingstein et al., 2020; Walker et al., 2021). Thus, any enhanced loss of CO_2_ *via* SOC decomposition has significant implications for global warming (Zhang et al., 2020).

Anthropogenic nitrogen (N) inputs are reported to significantly affect SOC content (Mazzoncini et al., 2011; Riggs and Hobbie, 2016; Chen et al., 2021). Globally, anthropogenic N inputs have increased from 156 Tg N yr^–1^ in 1995 to 193 Tg N yr^–1^ in 2010 (Galloway et al., 2008; Fowler et al., 2015), and it is estimated that by 2050 the global rate of N inputs will double the rate in 1995 (Penuelas et al., 2020). A considerable portion of the anthropogenically derived N is transformed into nitrate, which can leach to depth (>1 m) within the soil profile (Van Meter et al., 2016; Xin et al., 2019; Yang et al., 2020; Gao et al., 2021). As the soil profile deepens, persistent hypoxia or even anoxia can establish, potentially resulting in nitrate being reduced *via* the denitrification or dissimilatory nitrate reduction to ammonium (DNRA) pathways, which require SOC as an electron donor (Laursen and Seitzinger, 2002; Giblin et al., 2013). Consequently, anthropogenic N inputs potentially affect SOC decomposition not only at the soil surface but also in the deeper soil profile.

The SOC in deep soil is expected to respond to N addition differently from that of surface soil due to carbon sources being distinctively different between the surface soil and deep soil (Salomé et al., 2010). In surface soil, plant residues and root exudates are important sources of SOC. In line with this, increased CO_2_ emission following N addition were found to be derived from plant residues and root exudates (Schulte-Uebbing and de Vries, 2018; Xu et al., 2021). This mechanism would be expected to be less significant in deep soil since the contribution of plant residue and roots to SOC sharply decreased with the increasing soil depth (Poirier et al., 2018). Furthermore, oxygen (O_2_), an electron acceptor for SOC oxidation, is more available to SOC decomposers in surface soil than in deep soil. The soil O_2_ concentration generally declines sharply from the surface to a depth of approximately 1.0 m, then continues to decline slowly with the increase of soil depth (Sierra and Renault, 1998; Orem et al., 2011; Kautz, 2015). Thus, nitrate in deep soil has a larger opportunity to replace O_2_ as an alternative electron acceptor for SOC oxidation. Compared with the SOC in surface soil, the SOC in deep soil is generally absorbed or co-precipitated with minerals as mineral associated organic carbon (MAOC), which potentially decreases its accessibility for soil microbial decomposition (Stuckey et al., 2018; Jilling et al., 2021). The observed increase in decomposers, induced by N addition in deep soil, is expected to enhance the potential for decomposers to destabilize MAOC (Feng et al., 2022).

Thus, the response of the carbon following N addition differs since the distinct SOC in surface and deep soils. Most previous studies have only investigated the response of SOC following N addition in the surface soil (<1.0 m depth). Many of these studies reported that N addition increased SOC content (Riggs et al., 2015; Philben et al., 2019), while other studies reported that N addition decreased (Mo et al., 2008; Treseder, 2008; Bulseco et al., 2019) or did not affect SOC content (Högberg, 2007; Lu et al., 2011), this may be attributed to the form of N, the level of application and soil type. While, few studies have investigated the effects of N addition on carbon decomposition in deep soil (Li et al., 2014; Xu et al., 2021). Such information is relevant for understanding MAOC stability in the deep soil and carbon sequestration.

In this study, we investigated the responses and relevant mechanisms of MAOC stability following nitrate addition to deep soil. Nitrate was selected as the N source because it is the main form of anthropogenic N that leaches into deep soil.

## 2. Materials and methods

### 2.1. Experimental site and sample collection

Soil samples were collected from the campus of the Fujian Agriculture and Forestry University, Fuzhou, China (26°06′ N, 119°13′ E). Three depths of soil (1.5–1.7, 2.0–2.2, and 2.5–2.7 m) were collected. Deep soil in this study is defined as soil depths >1.0 m. The soil samples were passed through a 2 mm sieve to remove as much live and dead root material, then basic soil physicochemical properties were determined, which are shown in Table 1. Soils were placed in sealed ziplock bags, with excess air removed using vacuum package machine to minimize exposure to O_2_, and stored at −20°C about 3 days, then we started the experiments. Soils were thawed at 4°C and preincubated at 20°C (Fontaine et al., 2007; Condron et al., 2014) for 5 days under anaerobic condition to recover microbial activity prior to commencing experiments.

**TABLE 1:** The basic physicochemical properties of the soil.

		Soil depth	
	1.5–1.7 m	2.0–2.2 m	2.5–2.7 m
SOC (g C kg^–1^ dry soil)	4.25 ± 0.09a	4.14 ± 0.01a	3.50 ± 0.04b
DOC (mg C kg^–1^ dry soil)	73.81 ± 11.78a	68.33 ± 5.75a	59.69 ± 6.19a
NO_3_^–^ (mg N kg^–1^ dry soil)	3.09 ± 0.19b	11.20 ± 0.26a	2.22 ± 0.21c
NO_2_^–^ (mg N kg^–1^ dry soil)	1.14 ± 0.06b	2.20 ± 0.48a	1.34 ± 0.08b
NH_4_^+^ (mg N kg^–1^ dry soil)	15.26 ± 0.73a	16.91 ± 3.63a	14.85 ± 0.90a
Moisture content	19.98%	26.96%	23.87%
EC (mS cm^–1^)	79.1	94.5	50.9
pH	5.17	5.05	5.15
Sand (%)	69.13	59.62	46.16
Silt (%)	29.27	37.46	48.47
Clay (%)	1.6	2.92	5.36

Different letters indicate significant differences (*P* < 0.05) among the soil depths.

The SOC content was determined using an elemental analyzer (Vario Macro Cube, Elementar, Germany). Soil moisture content was determined by drying fresh soil samples to constant weight at 105°C oven. Soil samples were extracted with 1 M KCl solution (soil: liquid ratio was 1:5) and then filtered (0.45 μm, Jinteng, China). The soil extracts were then analyzed for dissolved organic carbon (DOC) concentration using a total organic carbon analyzer (TOC-LCPH, Shimadzu, Japan), for nitrate, nitrite and ammonium concentrations using a UV-1800 spectrophotometer (Shimadzu, Japan) and the colorimetric method (Norman and Stucki, 1981; Dorich and Nelson, 1983; Norman et al., 1985), and for pH using a pH meter (LeiCi PHSJ-3F, China). After extracting soil samples with distilled water (soil: water ratio was 1:5) and filtering, the electrical conductivity (EC) was determined with a conductivity meter (LeiCi DDSJ-308F, China). Soil texture was determined using a laser particle analyzer (Malvern Mastersizer 3000, UK) according to the protocol (Pieri et al., 2006).

### 2.2. Experimental design

#### 2.2.1. Experiment 1: Effects of nitrate addition on deep soil respiration, microbial community structure and key functional genes responsible for C degradation

In order to determine the effect of nitrate addition on CO_2_ emission from deep soil, two treatments were conducted: (1) nitrate addition treatment: 15 g of fresh soil was placed in to 120 ml flasks and a KNO_3_ solution was added to the flasks to reach 100 mg NO_3_^–^-N kg^–1^ dry soil; (2) control treatment: 15 g fresh soil received an equal amount of distilled water. Moisture is reported to be the most important factor affecting SOC mineralization (Huang and Hall, 2017). Thus, three gravimetric water contents were applied: 35% (2 ml 50 mM KNO_3_), 70% (5 ml 20 mM KNO_3_), and 200% (20 ml 5 mM KNO_3_). There was a total of 54 flasks (2 treatments × 3 soil depths × 3 soil moisture contents × 3 replicates). All flasks were sealed with air-tight butyl rubber septa and aluminum caps. The headspace gas was alternatively evacuated (0.1 kPa) and flushed with pure helium (99.999%, 120 kPa) five times to remove O_2_ (Yuan et al., 2019), the initial O_2_ concentrations was 35.5 μmol L^–1^ at this time. All flasks were incubated at 20°C (Fontaine et al., 2007; Condron et al., 2014) in the dark for 55 days.

During the incubation, the headspace CO_2_ concentrations were periodically determined using a robotized sampling and analysis system (Molstad et al., 2007). Briefly, the robotized system consisted of an incubation system linked with a gas collection and analysis system. It enabled sampling of the headspace gas by puncturing the butyl rubber septa of the anaerobic flasks and then pumping of 2 ml sample gas through the loop of the GC with a peristaltic pump. An electron capture detector (ECD) was used for determination of N_2_O and a thermal conductivity detector (TCD) was used to measure CO_2_, O_2_ and N_2_.

At the end of the 70% water content incubation, soil samples from the nitrate addition and control treatments, for each soil depth, were collected to determine the soil microbial community structure, and the key functional genes responsible for C degradation. Soil microbial DNA was extracted from these samples using the PowerSoil DNA isolation kit (MoBio, Carlsbad, CA) according to the manufacturer’s instructions. V3-V4 variable region of the 16S rRNA gene were amplified with primers 338F (ACTCCTACGGGAGGCAGCAG)/806R (GGACTACHVGGGTWTCTAAT). The sequencing operation was completed by Beijing Allwegene technology Co., Ltd. (Beijing, China). Sample sequences were clustered with a threshold of 97% similarity to obtain representative operational taxonomic units (OTUs). Paired-end sequencing was performed using an Illumina Miseq PE300 platform (Wu et al., 2019).

The total RNA was extracted from 1 g soil samples using the RNA Extraction Kit (Tiangen Biochemical Science Technologies Co., Ltd., Beijing, China) according to the manufacturer’s protocols. The RNA concentration and purity were determined using an ND-2000 spectrophotometer (Thermo Scientific), then RNA integrity was assessed using a Tanon 1600 imaging system (Tanon Science and Technology Co., Ltd., Shanghai, China). The primers were synthesized by Invitrogen (Shanghai, China), subsequently, RNA was converted to cDNA using the Prime Script™ RT reagent Kit with gDNA Eraser (TaKaRa). Then quantitative Real-Time PCR (qRT-PCR) was performed using an ABI7500 quantitative PCR system (Applied Biosystems, USA) with each sample conducted in triplicate. The relative abundances of genes responsible for the degradation of starch, hemicellulose, cellulose, chitin, aromatics, lignin and lignin from labile to recalcitrant (*amyA*, *arA*, *cbhI*, *chi*, *AceB*, *lip*, and laccase-like multi-copper oxidase (*Lmco*), respectively) were determined using the 2^–ΔΔCt^ method (Wang et al., 2019), with the 16S rRNA gene used as an internal reference gene, the denitrification genes for qRT-PCR were *narG*, *nirK*, and *nosZ* genes. The primer sequences of qRT-PCR are presented in Supplementary Tables 1, 2.

#### 2.2.2. Experiment 2: Effects of supplemental amount of nitrate on deep soil CO_2_ emissions

We further tested whether the increase in soil CO_2_ emissions was linearly correlated with the supplemental rate of nitrate addition, using the soil sample from 2.0 to 2.2 m depth with a 70% water content, including the subsequent experiments. The reason for continuing using 2.0–2.2 m depth was the higher level of nitrate concentration in this layer than other layers and the reason for continuing using 70% moisture content was more reasonable and a real condition to explore the mechanism. Five nitrate levels were applied: 0, 10, 20, 50, and 100 mg NO_3_^–^-N kg^–1^ dry soil. Each level was replicated three times and flasks were incubated in the dark at 20°C for 98 days. The CO_2_ concentration was determined every 7 days and analysis methods were identical to that used in Experiment 1.

#### 2.2.3. Experiment 3: Effects of ammonium addition on deep soil CO_2_ emissions

We further tested if other inorganic N types beside nitrate, e.g., ammonium, could promote deep soil CO_2_ emissions. Three treatments were applied, (1) nitrate addition treatment: 15 g fresh soil of the 2.0–2.2 m depth was cultured in 120 ml flasks with 5 ml of 20 mM KNO_3_ (the final nitrate content was 100 mg NO_3_^–^-N kg^–1^ dry soil); (2) ammonium addition treatment: 15 g fresh soil of the 2.0–2.2 m depth was cultured in 120 ml flasks with 5 ml of 20 mM NH_4_Cl (the final ammonium content was 100 mg NH_4_^+^-N kg^–1^ dry soil); (3) control treatment: 15 g fresh soil of the 2.0–2.2 m depth received 5 ml of distilled water. The flasks were incubated in the dark at 20°C for 98 days and headspace gas sampling occurred every 7 days.

#### 2.2.4. Experiment 4: Effects of O_2_ level on the stimulation of nitrate on deep soil CO_2_ emissions

We further determined if nitrate acted as an alternative electron acceptor to O_2_ in stimulating deep soil CO_2_ emission. The initial O_2_ concentration in the flasks was set at 1% by volume. During the incubation, the O_2_ concentration was expected to gradually decrease. Two treatments were set up: (1) 1% O_2_ treatment: 15 g fresh soil from the 2.0 to 2.2 m depth was incubated with 5 ml of distilled water in 120 ml flasks; (2) 1% O_2_ + NO_3_^–^ treatment: 15 g fresh soil from the 2.0 to 2.2 m depth was incubated with 5 ml of 20 mM KNO_3_ solution in 120 ml flasks. The headspace of the flasks was alternatively evacuated (0.1 kPa) and re-flushed with high-purity helium/O_2_ mixture gas (1% O_2_ and 99% helium, 120 kPa) five times, the initial O_2_ concentrations was 565 μmol L^–1^ at this time, and supplemented with 1% O_2_ again when O_2_ concentrations declined below 100 μmol L^–1^. A total of 96 flasks were prepared (48 flasks for each treatment) and incubated under dark conditions at 20°C for 98 days. At the beginning of the incubation, three flasks from each treatment were randomly selected for periodically determining the headspace O_2_ and CO_2_ concentrations at a frequency of four measurements per month, using the robotized sampling and analyzing system as noted above in Experiment 1. To calculate the stoichiometric mole ratio of nitrate and oxygen when nitrate was used as an electron acceptor, during the incubation, three flasks of each treatment were randomly selected each week to destructively sample the soil for determining the nitrate concentrations.

#### 2.2.5. Experiment 5: Effects of nitrate addition on microbial biomass N and C contents, MAOC, and redox potential in deep soil

We further tested whether the promotion of microbial respiration by nitrate would stimulate microbial proliferation and consequently increase the microbial utilization of on MAOC in deep soil. Two treatments were conducted: (1) 15 g soil samples from the 2.0 to 2.2 m depth were incubated with 5 ml of 20 mM KNO_3_; (2) 15 g soil samples from the 2.0 to 2.2 m depth were incubated with 5 ml of distilled water. A total of 54 flasks (27 flasks for each treatment) were prepared and their headspaces were exchanged with high-purity helium as described in Experiment 1. The flasks were incubated in the dark at 20°C for 98 days. Three flasks for each treatment were randomly selected every 14-day for destructive sampling to determine the MAOC content using the citrate-bicarbonate-dithionite (CBD) method (Lalonde et al., 2012). At the end of the incubation, three flasks from each treatment were used to measure the microbial biomass carbon (MBC) and nitrogen (MBN) using the fumigation-extraction method (Vance et al., 1987) and perform 16S DNA gene copy numbers together to estimate microbial proliferation. The last three flasks for each treatment were used to measure soil redox potential (Eh) using an Eh meter (Model HLY-216, China) and pH by using the probe inserted into the soil.

### 2.3. Statistical analysis

The statistical package SPSS 24.0 (SPSS Inc., Chicago, IL, USA) was used to perform all statistical analysis. Analysis of variance (ANOVA) was used to determine the difference (*P* < 0.05) among treatments after the Shapiro–Wilk and Levene tests were used to confirm the normality and homogeneity of the data.

## 3. Results and discussion

### 3.1. Nitrate addition promote microbial respiration in deep soil by acting as an alternative electron acceptor to O_2_

**FIGURE 1:**
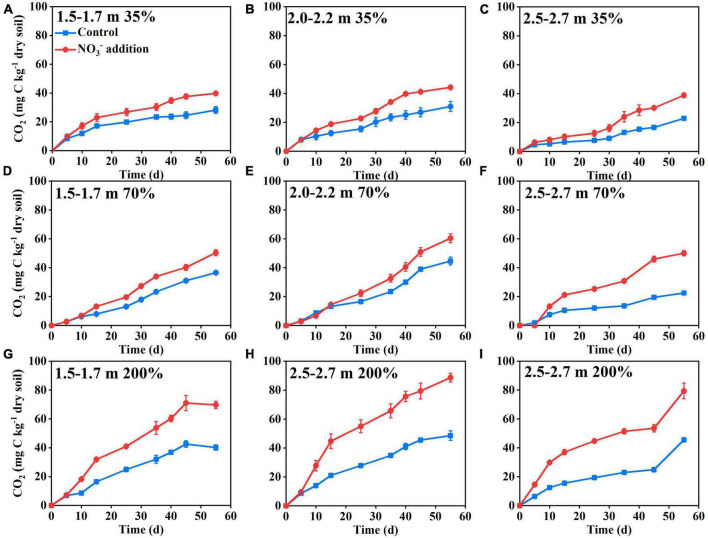
Nitrate addition effects on the cumulative CO_2_ emissions from deep soil depths: 1.5–1.7 m **(A,D,G)**, 2.0–2.2 m **(B,E,H)**, and 2.5–2.7 m **(C,F,I)** with soil gravimetric water contents of 35% **(A–C)**, 70% **(D–F)**, and 200% **(G–I)** in Experiment 1. The blue lines and red lines represent control and nitrate addition treatments, respectively. Data are shown as the mean ± standard deviation (*n* = 3).

**FIGURE 2:**
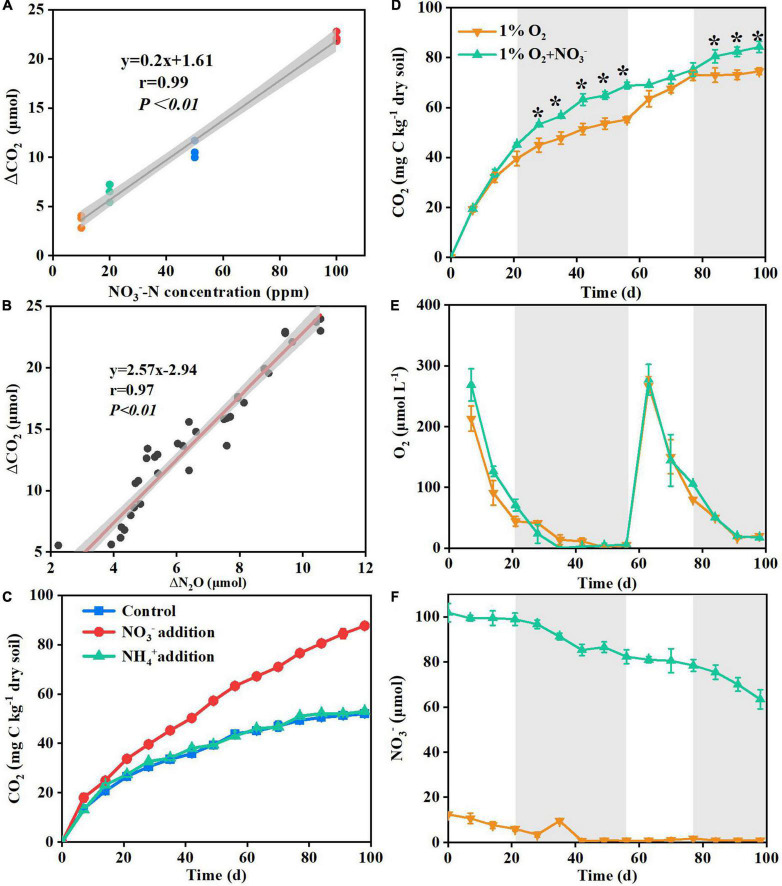
Correlation between the rate of the supplemented nitrate and the increasing concentration of ΔCO_2_ under the nitrate addition treatment in Experiment 2 **(A)**; correlation between the increasing amounts of the produced ΔCO_2_ and ΔN_2_O at 100 ppm NO_3_^–^-N treatment in Experiment 2 **(B)**; ammonium versus nitrate addition effects on the cumulative CO_2_ emissions from deep soil in Experiment 3 **(C)**; dynamics of CO_2_ **(D)**, O_2_ **(E)** and NO_3_^–^ **(F)** concentrations in flasks under 1% O_2_ and 1% O_2_ + NO_3_^–^ treatments in Experiment 4. Delta indicates the value of nitrate addition treatment minus non-nitrate control within each sampling day. The gray areas in panels **(A,B)** indicate 95% confidence intervals. The gray areas in panels **(D–F)** indicate anaerobic conditions with O_2_ concentrations below 100 μmol L^– 1^. Asterisk denotes significant difference (*P* < 0.05) between the two treatments. Data are shown as the mean ± standard deviation (*n* = 3).

The results of Experiment 1 showed that there was little difference in the cumulative CO_2_ emissions from deep soil between the nitrate addition treatment and the control treatment during the initial 10 days of incubation (Figure 1). With increasing incubation time, the cumulative CO_2_ emissions differed significantly between the two treatments (Figure 1). At the end of Experiment 1 (55 days of incubation), the cumulative CO_2_ emissions under the nitrate addition treatment had increased 40–140% relative to the control treatment, with the increase dependent on soil water moisture content and soil depth (Supplementary Figure 1). The 200% water content significantly contributed to ΔCO_2_ at three depths compared to the 35 and 70% water contents, soil moisture affects the various life activities of soil microorganisms, under low soil moisture conditions microbial activity may be limited, while increased moisture could significantly enhance microbial activity, leading to an improvement in soil respiration. Compared to depths 1.5–1.7, 2.0–2.2, and 2.5–2.7 m depth had higher ΔCO_2_ at 35, 70, and 200% water content, reaching 60, 120, and 150%, respectively, indicating a higher sensitivity for the deeper soils. These results demonstrate that nitrate addition stimulated the microbial respiration in the deep soil depths under anaerobic conditions. Results of Experiment 2, where the increase in CO_2_ emission was significantly correlated (*P* < 0.01) with the nitrate addition rate (Figure 2), also support this.

There was a lag in the CO_2_ emission response to nitrate addition during the incubation (Figure 1). This lag was probably caused by the residual O_2_ in the soil pores which removed the need for nitrate to be used as an alternative electron acceptor (Parkin and Tiedje, 1984; Song et al., 2019). This was tested by monitoring the responses of soil respiration to varying O_2_ concentration. The results showed that the promoting effects of nitrate addition on soil respiration appeared when the headspace O_2_ concentration was below 100 μmol L^–1^, then disappeared after the injection of additional O_2_, and finally re-appeared after the O_2_ concentration was again below 100 μmol L^–1^ (Figure 2). By calculation, we found that the role of nitrate was activated when the stoichiometric mole ratio of nitrate to O_2_ exceeded 6:1 (77.4 μmol/12.7 μmol). Further evidence supporting the effect of nitrate in promoting soil respiration was the mole ratio of the CO_2_ to N_2_O produced under the nitrate addition treatment, which was 2.57:1 and close to the theoretical mole ratio 2:1 (Mørkved et al., 2006) when nitrate is used as an electron acceptor for microbial respiration (Figure 2).

Apart from acting as an alternative electron acceptor for microbial respiration, nitrate is a key N source for soil microbes (Geisseler et al., 2010; Wang et al., 2015). Previous studies have shown that N addition can promote surface soil respiration by serving as a nutrient (Soong et al., 2018). In order to test whether the positive effects of nitrate on soil respiration were caused as the result of enhanced N supply, equal amounts of nitrate-N or ammonium-N were added into the 2.0–2.2 m depth soil in Experiment 3. The results showed that, contrary to the nitrate-N treatment, the ammonium-N treatment did not significantly increase soil respiration (Figure 2). These results indicated that deep soil respiration could not be facilitated by merely changing the soil N status without acting as an electron acceptor. Briefly, above results suggested that the positive effects of nitrate on deep soil respiration were the result of it acting as an electron acceptor.

### 3.2. The enhancement of microbial respiration by nitrate promotes microbial growth and consequently destabilize MAOC in deep soil

**FIGURE 3:**
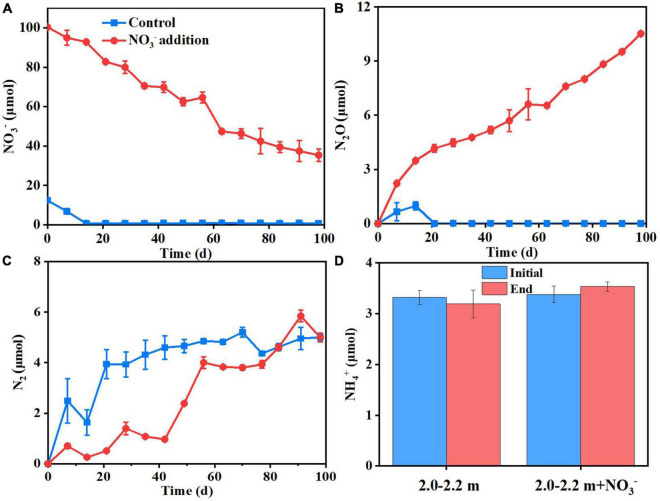
Dynamics of NO_3_^–^ **(A)**, N_2_O **(B)**, and N_2_ **(C)** concentrations in flasks under control and nitrate addition treatments. NH_4_^+^ concentrations at initial and end of the incubation under two treatments **(D)**.

**FIGURE 4:**
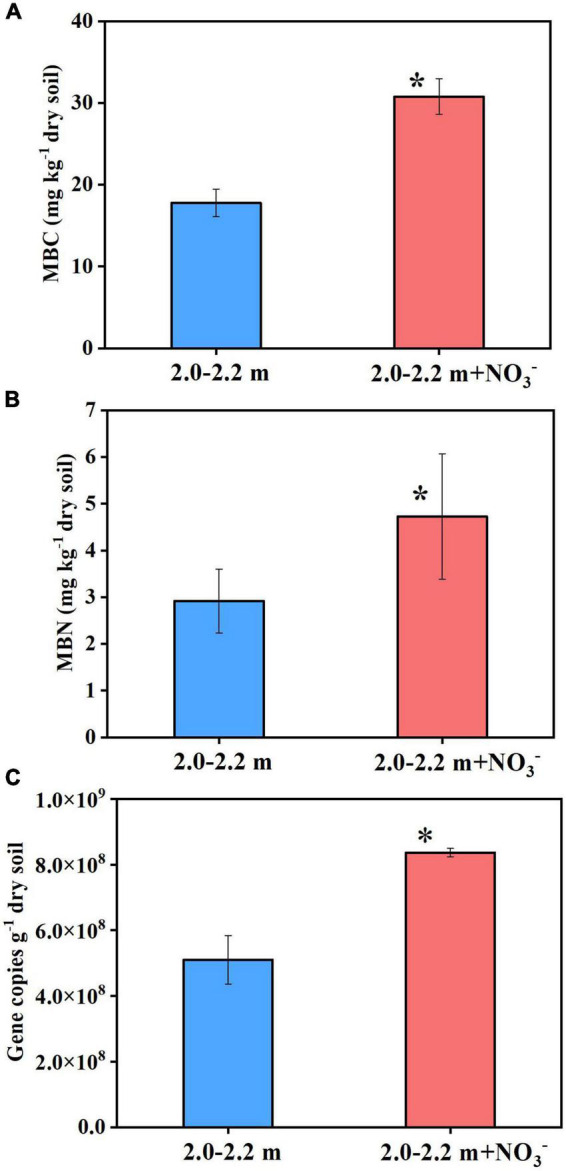
Nitrate addition effects on the MBC **(A)**, MBN **(B)**, and 16S DNA gene copies **(C)** in deep soil after 98 days at the end of Experiment 5. Data are shown as the mean ± standard deviation (*n* = 3). Asterisk denotes significant difference (*P* < 0.05) between the two treatments.

The increase in microbial access to an electron acceptor for respiration following nitrate addition tends to promote microbial assimilation and reproduction (Dyckmans et al., 2006). As Figure 3 shows, the soil ammonium concentration did not change significantly (*P* > 0.05) between the beginning and end of the incubations, which indicated that dissimilatory nitrate reduction to ammonium (DNRA) was negligible. In addition, study showed that DNRA may be a minimal pathway at high nitrate concentrations (Handler et al., 2022), it is generally believed that low nitrogen and high carbon will tilt the balance to DNRA (Van Den Berg et al., 2016; Pandey et al., 2020; Wei et al., 2022), the opposite is the high nitrogen and lower carbon contents in this study. Denitrification was the main nitrate reduction pathway, the amount of nitrate consumed (65 μmol N) was significantly larger than the cumulative amount of the N_2_O plus N_2_ (31 μmol N) produced by day 98 (Experiment 2). This indicates that about ∼50% of the added nitrate could have been assimilated by microbes for cell proliferation. In deep soils, microorganisms are expected to be in short supply of both C and N, because the microbial available C and N species, such as glucose, nitrate and ammonia, generally decrease sharply from the surface soil to deep soil. Consequently, the nitrate addition is expected to relief the microbial N starvation in deep soil and in turn promote the microbial growth there. In support of this were the measured increases (*P* < 0.05) in microbial DNA concentration (Supplementary Figure 2), MBC and MBN, and 16S DNA gene copy numbers (Figure 4) under nitrate addition relative to the control treatment at the end of Experiment 1 and 5. Thus, nitrate addition promoted microbial growth.

**FIGURE 5:**
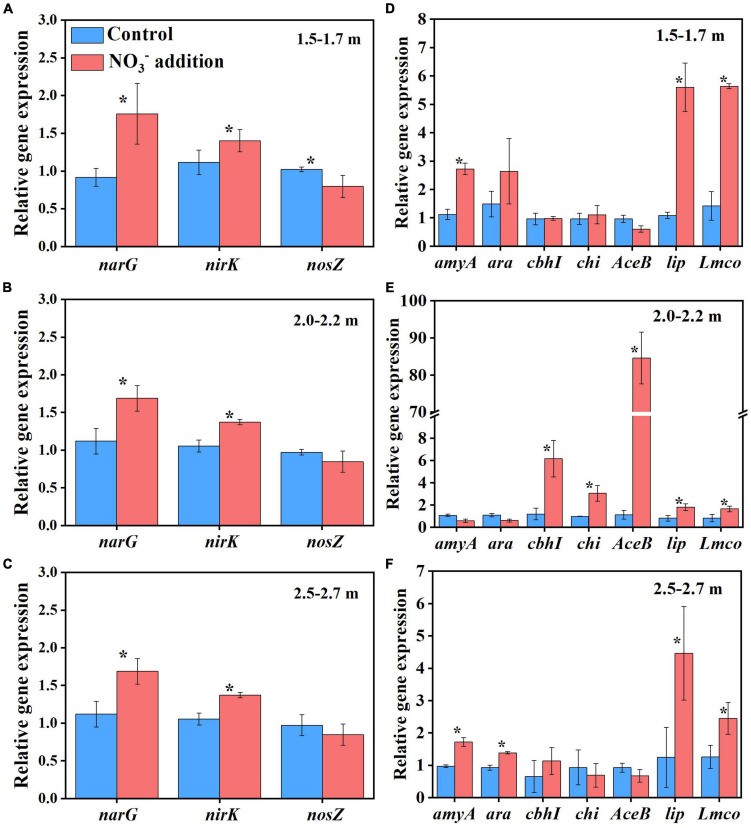
Nitrate addition effects on the relative gene abundances of denitrification genes (*narG*, *nirK*, and *nosZ*) and key and recognized C degradation genes (*amyA*, *arA*, *cbhI*, *chi*, *AceB*, *lip*, and *Lmco*) in deep soil depths of 1.5–1.7 m **(A,D)**, 2.0–2.2 m **(B,E)**, and 2.5–2.7 m **(C,F)** after 55 days at the end of Experiment 1. Data are shown as the mean ± standard deviations (*n* = 3). Asterisk denotes significant difference (*P* < 0.05) between the two treatments.

Apart from increasing microbial biomass, the nitrate addition treatment in Experiment 1 also significantly changed the soil microbial community composition (Supplementary Figure 2). Compared with the control treatment, nitrate addition significantly increased the relative abundances of *Bacillus*, *Aquabacterium*, *Sediminibacterium*, and *Acidibacter* at the genus level across all depths, and *Caproiciproducens* at 1.5–1.7 and 2.0–2.2 m (Supplementary Figure 3). It has been suggested that *Bacillus* and *Aquabacterium* contribute to denitrification in terrestrial and possibly other ecosystems (Verbaendert et al., 2011; Zhang et al., 2016). In addition, *Bacillus* and *Aquabacterium* were previously reported to play a key role in accelerating SOC decomposition (Lin et al., 2019; Yin et al., 2019). *Caproiciproducens* genus could accelerate the use of carbon sources for conversion to CO_2_ (Kim et al., 2015). In this study, the amounts of CO_2_ and N_2_O emitted were correlated with the relative abundances of *Bacillus*, *Aquabacterium* and *Sediminibacterium* (Supplementary Figure 4), indicating that these microbes could have contributed to the positive effects of nitrate addition on deep soil respiration. In addition, the expressions of functional genes of *narG* and *nirK* under the nitrate addition treatment was significantly (*P* < 0.05) higher than control treatment, while the *nosZ* gene was not significantly different between the two treatments (*P* > 0.05) at 2.0–2.2 and 2.5–2.7 m depths except for a decrease for the nitrate addition treatment at 1.5 m depth (Figure 5).

**FIGURE 6:**
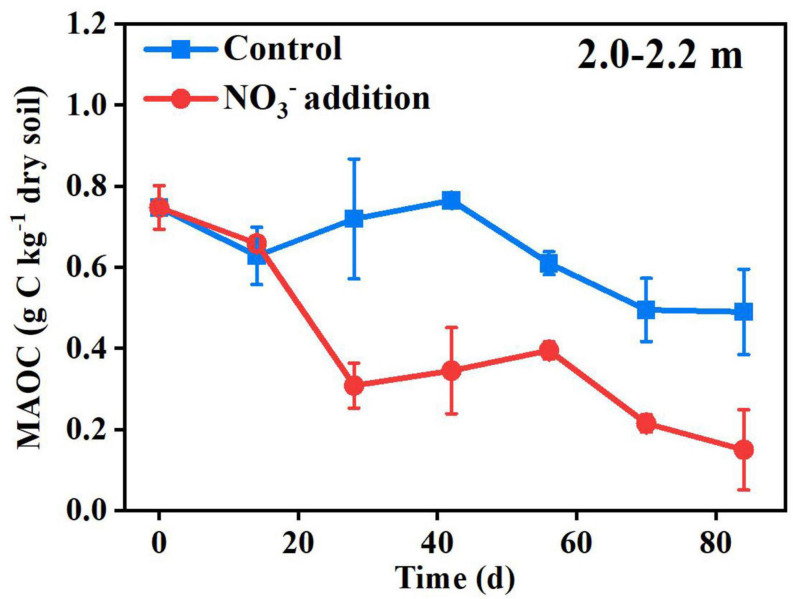
Nitrate addition effects on the content of MAOC in the 2.0–2.2 m depth over time in Experiment 5. Data are shown as the mean ± standard deviations (*n* = 3).

The SOC in deep soils is generally bound to soil minerals, which protect SOC from microbial attack (Han et al., 2016; Gartzia-Bengoetxea et al., 2020). Previously, it was reported that the fluctuation of pH and Eh may also cause solubilization of MAOC, with the solubilization rapidly activated when the Eh decreased below 150 mV (Grybos et al., 2009). In this study, the pH and Eh were not significantly different (*P* > 0.05) between the control treatment (5.09 ± 0.07 and 156.67 ± 4.04 mV) and the nitrate addition treatment (5.15 ± 0.06 and 153.00 ± 4.36 mV) at the end of incubation (Supplementary Figure 5), indicating that the pH and Eh were not responsible for the difference in MAOC between the two treatments. On the contrary, the expressions of functional genes typically responsible for carbon decomposition, such as *amyA* at 1.5–1.7 and 2.5–2.7 m, *AceB* at 2.0–2.2 m and *lip* and *Lmco* at all three depths were significantly greater (*P* < 0.05) under the nitrate addition treatment than under the control treatment at the end of Experiment 1 (Figure 5). The increases in carbon decomposer abundance, as noted above, and their functional gene expression were previously reported to increase the microbial utilization of MAOC (Li H. et al., 2021). Our results show that the content of the MAOC under the nitrate addition treatment was significantly lower than that under the control treatment from as early as day 28 of the incubation, and the ratio of MAOC/SOC decreased from 20% before incubation to 4% at the end of incubation (Figure 6), which is in accordance with previous studies reporting that N addition not only modified the composition and abundance of bacteria, but also decreased the MAOC complexes (Qin et al., 2020; Li J. et al., 2021). These results indicate that the increase in microbial utilization of MAOC, under the nitrate addition treatment, destabilizes the MAOC.

## 4. Conclusion

This study demonstrated that nitrate acted as an alternative electron acceptor to O_2_ for microbial respiration and consequently promoted the growth of SOC decomposers in deep soil (depths > 1 m). The increases in SOC decomposer abundances and functional genes known to align with SOC decomposition in turn increased the microbial utilization of the MAOC, resulting in the acceleration of SOC decomposition in deep soil. Our results have implications for understanding the contribution of deep SOC to atmospheric CO_2_ in response to anthropogenic reactive N enrichment of the environment. According to the results of this study, increased nitrate leaching under anaerobic conditions will enhance the decomposition of MAOC in deep soil. Since the promoting effects of nitrate on soil respiration is derived from its role as alternative respiration acceptor to O_2_, the potential of nitrate to destabilize MAOC is expected to be favored in deep soils with clay texture and higher water content. Consequently, reducing nitrate leaching will assist in preserving MAOC in deep soil.

## Data availability statement

The data presented in this study are deposited in the NCBI repository, accession number: PRJNA911917.

## Author contributions

WS: methodology, visualization, formal analysis, and writing – original draft. CH, YL, TC, NW-M, TG, and JL: review and editing. SZ: supervision and review and editing. SQ: funding acquisition, conceptualization, and writing – review and editing. All authors read and approved the final manuscript.
