# Supplementary material for: Nitrate as an alternative electron acceptor destabilizes the mineral associated organic carbon in moisturized deep soil depths
Source: Front Microbiol. 2023 Feb 8;14:1120466. doi: 10.3389/fmicb.2023.1120466 (PMC9944454; doi:10.3389/fmicb.2023.1120466)
Supplement: Supplementary file 1 [file Data_Sheet_1.docx]

**Supplemental Information**

Table. S1 Main functional genes of carbon degradation and qRT-PCR primer sequences

| *Genes* | Carbon degradation | Primer sequences |
| --- | --- | --- |
| *amyA* | Starch | TGCCCACCCCTGGTTCAAAT **and** GGGTTCCTGCTCCCAGATGC |
| *ara* | Hemicellulose | AACGCCATCCACGCTAAATA **and** ACGGCATACTCGCCTAACAA |
| *cbhI* | Cellulose | ACCAA[C,T]TGCTA[C,T]ACI[A,G]G[C,T]AA **and** GC[C,T]TCCCAIAT[A,G]TCCATC |
| *chi* | Chitin | GATGTGGCAAAGGGCTACTA **and** TTCCAATCAGCGAGTCAGTT |
| *AceB* | Aromatics | CTGCTGACCCTGCCCCTGAT **and** GATGTGCTGGCTGGAAATGC |
| *lip* | Lignin | GTCCTTCGTCGTCCCAGGAGCCACGTTCC **and** GGGAGTNGAGTCGAAGGG |
| *Lmco* | Lignin | ACMWCBGTYCAYTGGCAYGG **and**  TGICCRTGIARRTGIANIGRTG |

Table. S2 PCR primers for *nirK*, *nosZ* and *narG* genes

| *Genes* | Primer sequences |
| --- | --- |
| *nirK* | ATCATGGTSCTGCCGCG **and**  GCCTCGATCAGRTTGTGGTT |
| *nosZ* | CGYTGTTCMTCGACAGCCAG **and**  CGSACCTTSTTGCCSTYGCG |
| *narG* | TAYGTSGGGCAGGARAAACTG **and**  CGTAGAAGAAGCTGGTGCTGTT |

**A**

**B**

**C**

**Fig. S1** The ΔCO_2_ from deep soil depths of 1.5-1.7 m (**A**), 2.0-2.2 m (**B**) and 2.5-2.7 m (**C**) under 35%, 70% and 200% water contents in Experiment 1. The ΔCO_2_ (%) was calculated by formula: ΔCO_2_ (%) = (nitrate addition treatment value minus non-nitrate control value)/ non-nitrate control value × 100. Data were shown as the mean ± standard deviation (n=3).

**Fig. S2** Nitrate addition effects on the microbial DNA concentrations in deep soils after 55 days at the end of Experiment 1. Data are shown as the mean ± standard deviation (n=3). Asterisk denotes significant difference (*P<0.05*) between the two treatments.


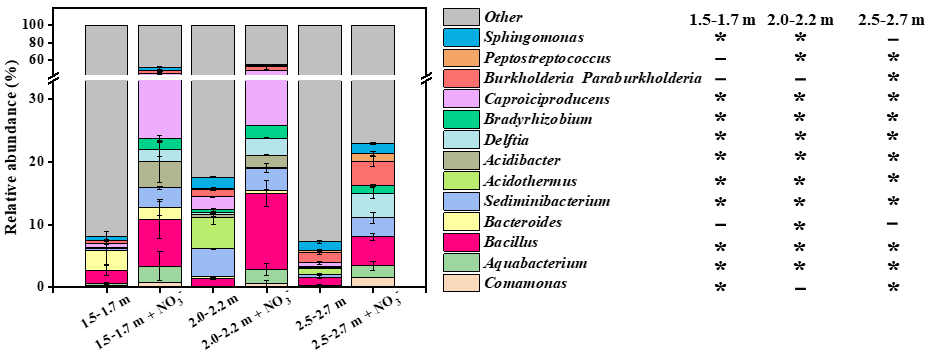


**Fig. S3** Nitrate addition effects on soil microbial community composition at the genus level at the end of the incubation in Experiment 1. Asterisk indicates significant differences (*P<0.05*) between the two treatments under the same depth, the minus indicates no difference. Error bars indicate standard deviation (n=3)

**A**

**B**

**C**

**D**

**E**

**F**

**Fig. S4** Correlations between CO_2_ (**A**, **B** and **C**) and N_2_O (**D**, **E** and **F**) emission amounts and the relative abundance at genus level.

**A**

**B**

**Fig. S5** Nitrate addition effects on the pH (**A**) and Eh (**B**) at the end of Experiment 5. Data are shown as the mean ± standard deviation (n=3).
